# Parecoxib mitigates lung ischemia-reperfusion injury in rats by reducing oxidative stress and inflammation and up-regulating HO-1 expression

**DOI:** 10.1590/ACB360901

**Published:** 2021-10-25

**Authors:** Jun Qin, Xunling Su, Xin Jin, Jiayi Zhao

**Affiliations:** 1 Master. Department of Anesthesiology - Zhejiang Hospital - Hangzhou, China.; 2 Master. Department of Anesthesiology - Zhejiang Hospital - Hangzhou, China.; 3 Master. Department of Anesthesiology - Zhejiang Hospital - Hangzhou, China.; 4 Master. Department of Anesthesiology - Zhejiang Hospital - Hangzhou, China.

**Keywords:** Lung, Reperfusion Injury, Oxidative Stress, Rats

## Abstract

**Purpose:**

To investigate the protective effect of parecoxib against lung ischemia-reperfusion injury (LIRI) in rats and the mechanism.

**Methods:**

Thirty rats were divided into sham-operated, LIRI and LIRI+parecoxib groups. LIRI model (ischemia for 60 min, followed by reperfusion for 120 min) was constructed in LIRI and LIRI+parecoxib groups. In LIRI+parecoxib group, 10 mg/kg parecoxib was given via femoral vein 15 min before ischemia beginning. At the end of the reperfusion, blood gas analysis, lung wet to dry mass ratio measurement, lung tissue biochemical determination and heme oxygenase-1 (HO-1) protein expression determination were performed.

**Results:**

Compared with LIRI group, in LIRI+parecoxib group the oxygenation index was significantly increased, the alveolar-arterial oxygen partial pressure difference was significantly decreased, the lung wet to dry mass ratio was significantly decreased, the lung tissue malondialdehyde content was significantly decreased, the lung tissue superoxide dismutase and myeloperoxidase activities were significantly increased, the lung tissue tumor necrosis factor α and interleukin 1β levels were significantly decreased, and the lung tissue HO-1 protein expression level was significantly increased (all P < 0.05).

**Conclusions:**

Parecoxib pretreatment can mitigate the LIRI in rats by reducing oxidative stress, inhibiting inflammatory response and up-regulating HO-1 expression in lung tissue.

## Introduction

Lung ischemia-reperfusion injury (LIRI) often occurs in lung transplantation, cardiopulmonary bypass, pulmonary embolism thrombectomy, isolated pulmonary perfusion and other surgeries^1^. It is a hot and difficult research topic in the field of cardiopulmonary vascular diseases. LIRI often develops to acute respiratory distress syndrome (ARDS), manifested by dyspnea, pulmonary edema, and hypoxemia. Despite the continuous improvement of medical technology, the clinical mortality of ARDS remains high^2^. At present, the prevention and treatment of LIRI are still very limited, and the pathogenesis of LIRI has not been fully clarified. During the ischemia-reperfusion, the neutrophils are activated and release the pro-inflammatory factors. This causes the apoptosis, necrosis and tissue injury, and eventually leads to the organ dysfunction^3^.

Cyclooxygenase (COX) is an important rate-limiting enzyme in the synthesis of prostaglandins. COX has two isozymes, including COX-1 and COX-2. COX-2 is an immediate early gene, which is highly expressed induced by many factors. It is mainly located in the nuclear membrane. The prostaglandins produced by COX-2 catalysis can preferentially enter the nucleus and regulate the transcription of target genes^4^. It is found that, under the ischemia-reperfusion injury, the level of COX-2 is increased. Inhibition of COX-2 expression can reduce the production of inflammatory factors and alleviate the ischemia-reperfusion injury^5^.

Parecoxib is a specific COX-2 inhibitor commonly used in clinics. It has been found that parecoxib can attenuate the hepatic ischemia-reperfusion injury in rats by inhibiting the inflammation and oxidative stress^6^. Heme oxygenase-1 (HO-1) is an inducible heme oxygenase, and its activation is one of the most important cytoprotective mechanisms during cell stress^7^. Parecoxib can induce the HO-1 expression in macrophages and vascular smooth muscle cells through reactive oxygen species (ROS)-dependent pathway^8^. Study has shown that the over-expression of HO-1 can reduce the LIRI and acute lung injury induced by endotoxin^9^. The mechanism may be related to its antioxidation, microenvironment stability maintenance, anti-apoptosis and anti-inflammation^10^.

In the present study, the protective effect of parecoxib against LIRI in rats was investigated, and the mechanism related to the oxidative stress, inflammation, and HO-1 expression were explored.

## Methods

This study was approved by the ethics committee of Zhejiang Hospital. All animal procedures followed the Principles of Laboratory Animal Care and were in accordance with the *Guide for the Care and Use of Laboratory Animals*, by the National Institutes of Health.

### Construction of LIRI model

Male specific-pathogen-free sprague dawley (SD) rats (260-280 g) were used for experiment. Before the experiment, the rats were fasted for 8 h and drank freely. The anesthesia was performed by intraperitoneal injection of 1% pentobarbital sodium, with dose of 40 mg/kg. The rats were fixed in supine position. A median neck incision was made to separate the trachea. The trachea was cut open. The endotracheal tube was inserted into the trachea, and was connected to the small animal ventilator for mechanical ventilation. The parameters of ventilator were as follows: tidal volume, 8 mL/kg; respiratory rate, 60 times/min; ratio of inhalation to exhalation, 1:2. An incision was made in the right groin, and the femoral artery and femoral vein were separated. A 24-gauge venous indwelling needle was placed. Then, the rats were transferred to the right decubitus position. The thoracotomy was performed through the left fifth intercostal space to separate the left hilum. A 100 U/kg heparin was injected through the femoral vein. After 5 min, the left pulmonary artery was clamped with a noninvasive vessel clamp for 60 min of ischemia. Then, the vascular clamp was released, and the blood perfusion was restored for 120 min.

### Grouping and treatment

Thirty rats were randomly divided into sham-operated group, LIRI group and LIRI+parecoxib group, 10 individuals in each group. In the sham-operated group, only the left pulmonary hilum was separated by thoracotomy, without occlusion of pulmonary artery. The complete LIRI model was established in LIRI group and LIRI+parecoxib group. In parecoxib group, at 15 min before pulmonary artery occlusion, 10 mg/kg parecoxib was given via femoral vein. In the LIRI group and LIRI+parecoxib group, equal volume of normal saline was given via femoral vein.

### Blood gas analysis

After 120 min of reperfusion, the blood sample was collected through the femoral artery. The blood gas indexes were detected by automatic blood gas analyzer. The partial pressure of oxygen (PaO_2_) and partial pressure of carbon dioxide (PaCO_2_) were recorded. The oxygenation index (OI) and alveolar-arterial oxygen partial pressure difference (PA-aO_2_) were calculated as follows: OI = PaO_2_ / fraction of inspired oxygen (FiO_2_); PA-aO_2_ = (atmospheric pressure - saturated water vapor pressure) × FiO_2_ - PaCO_2_ / 0.8 - PaO_2_.

### Measurement of lung wet to dry mass ratio

After the blood gas analysis, the rats were executed by cervical dislocation. The left pulmonary hilum was ligated, and the left lung tissue was cut off. The upper one third of lung tissue was taken, and the blood on the surface was rinsed off with normal saline. The liquid on the surface was dried by filter paper. The lung tissue was weighed on electronic balance to obtain the wet mass. Then, the lung tissue was dried in an oven at 65°C for 48 h to obtain the dry mass. The lung wet to dry mass ratio was calculated.

### Biochemical determination of lung tissue

The middle one third of left lung tissue was taken and homogenized with normal saline to obtain the homogenate. After centrifuging at 2,500 rpm for 15 min, the supernatant was obtained. The malondialdehyde (MDA) content, superoxide dismutase (SOD) activity and myeloperoxidase (MPO) activity were determined by spectrophotometer. The tumor necrosis factor a (TNF-a) and interleukin 1ß (IL-1ß) levels were determined by enzyme linked immunosorbent assay. The determination procedures were according to the instructions of the kits.

### Determination of HO-1 protein expression in lung tissue

The HO-1 protein expression in lung tissue was determined using western blotting assay. The lower one third of left lung tissue was taken and treated with 1-mL lysate. The total protein was extracted, and quantified by bicinchoninic acid method. A 50-µg crude protein sample was loaded on sodium dodecyl sulfate-polyacrylamide gel electrophoresis. The separated protein sample was transferred to nitrocellulose membranes, followed by blocking with 5% bovine serum albumin. The membranes were incubated with rabbit anti-rat HO-1 polyclonal antibody and rabbit anti-rat ß-actin polyclonal antibody at 4°C overnight, respectively. Then, the membranes were incubated with horseradish peroxidase-labeled IgG at room temperature for 2 h. Finally, the membranes were incubated with chemiluminescence reagent, followed by developing and scanning. The gray value of stripes on the membranes was measured using the gel image analysis system. The images were analyzed using ImageJ software. The ratio of gray value of HO-1 protein stripe to that of ß-actin stripe presented the expression level of HO-1 protein.

### Statistical analysis

All data were presented as the mean±SD. The statistical analysis was performed using Statistical Package for the Social Sciences (SPSS) software. The differences among three groups were assessed by one-way analysis of variance followed by post-hoc least significant difference (LSD)-t test. P < 0.05 was considered to indicate a statistically significant difference.

## Results

### Blood gas analysis results

After 120 min of reperfusion, the blood gas analysis results showed that, compared with sham-operated group, in LIRI and LIRI+parecoxib groups the OI was significantly lower (P < 0.05), and the PA-aO_2_ was significantly higher (P < 0.05). Compared with LIRI group, in LIRI+parecoxib group the OI was significantly higher (P < 0.05), and the PA-aO_2_ was significantly lower (P < 0.05) (Table 1).

**Table 1:** Comparison of OI and PA-aO_2_ in three groups.

Group	n	OI (mmHg)	PA-aO_2_ (mmHg)
Sham-operated	10	455.39±36.50	10.24±2.89
LIRI	10	398.17±22.21 a	19.82±3.21 a
LIRI+parecoxib	10	424.44±23.04 a b	15.37±4.04 a b
F		10.444	19.712
P		< 0.001	< 0.001

a P < 0.05 *vs*. sham-operated group;

b P < 0.05 *vs*. LIRI group; LIRI: lung ischemia-reperfusion injury; OI: oxygenation index; PA-aO_2_: alveolar-arterial oxygen partial pressure difference.

### Lung wet to dry mass ratio

**Figure 1:**
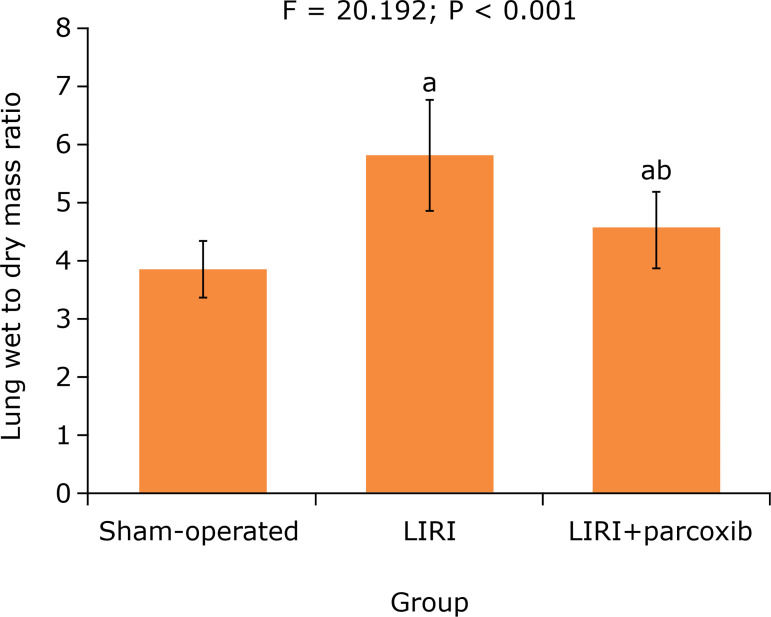
Comparison of lung wet to dry mass ratio in three groups (n = 10).

^a^P < 0.05 *vs*. sham-operated group; ^b^P < 0.05 *vs*. LIRI group; LIRI: lung ischemia-reperfusion injury.

After 120 min of reperfusion, in sham-operated, LIRI and LIRI+parecoxib groups, the lung wet to dry mass ratio was 3.82±0.48, 5.79±0.92 and 4.55±0.63, respectively. The lung wet to dry mass ratio in LIRI and LIRI+parecoxib groups was higher than that in sham-operated group (P < 0.05), but in LIRI+parecoxib group it was lower than in LIRI group (P < 0.05) (Fig. 1).

### Lung tissue MDA content and SOD and MPO activities

As shown in Table 2, at the end of reperfusion, compared with sham-operated group, in LIRI and LIRI+parecoxib groups the lung tissue MDA content was significantly increased (P < 0.05), and the lung tissue SOD and MPO activities were significantly decreased, respectively (P < 0.05). Compared with LIRI group, in LIRI+parecoxib group the lung tissue MDA content was significantly decreased (P < 0.05), and the lung tissue SOD and MPO activities were significantly increased, respectively (P < 0.05).

**Table 2:** Comparison of lung tissue MDA content and SOD and MPO activities in three groups.

Group	n	MDA (mol/mg)	SOD(U/mg)	MPO (U/g)
Sham-operated	10	0.72±0.12	14.21±2.02	2.20±0.16
LIRI	10	1.64±0.28 a	5.90±1.02 a	5.82±0.94 a
LIRI+parecoxib	10	0.97±0.20 a b	10.03±2.88 a b	3.39±0.53 a b
F		51.122	38.607	85.814
P		< 0.001	< 0.001	< 0.001

a P < 0.05 *vs*. sham-operated group;

b P < 0.05 *vs*. LIRI group; LIRI: lung ischemia-reperfusion injury; MDA: malondialdehyde; SOD: superoxide dismutase; MPO: myeloperoxidase.

### Lung tissue TNF-a and IL-1ß levels

At the end of reperfusion, the lung tissue TNF-a and IL-1ß levels in LIRI and LIRI+parecoxib groups were significantly higher than those in sham-operated groups, respectively (P < 0.05). Compared with LIRI group, each index in LIRI+parecoxib group was significantly decreased (P < 0.05) (Table 3).

**Table 3:** Comparison of lung tissue TNF-a and IL-1ß levels in three groups.

Group	n	TNF-a (pg/mg)	IL-1ß (pg/mg)
Sham-operated	10	273.65±56.18	465.19±77.20
LIRI	10	757.20±93.72 a	1403.83±174.94 a
LIRI+parecoxib	10	435.38±82.60 a b	785.42±135.33 a b
F		96.881	124.459
P		< 0.001	< 0.001

a P < 0.05 *vs*. sham-operated group;

b P < 0.05 *vs*. LIRI group; LIRI: lung ischemia-reperfusion injury; TNF-a: tumor necrosis factor a; IL-1ß: interleukin 1ß.

### Lung tissue HO-1 protein expression level

**Figure 2:**
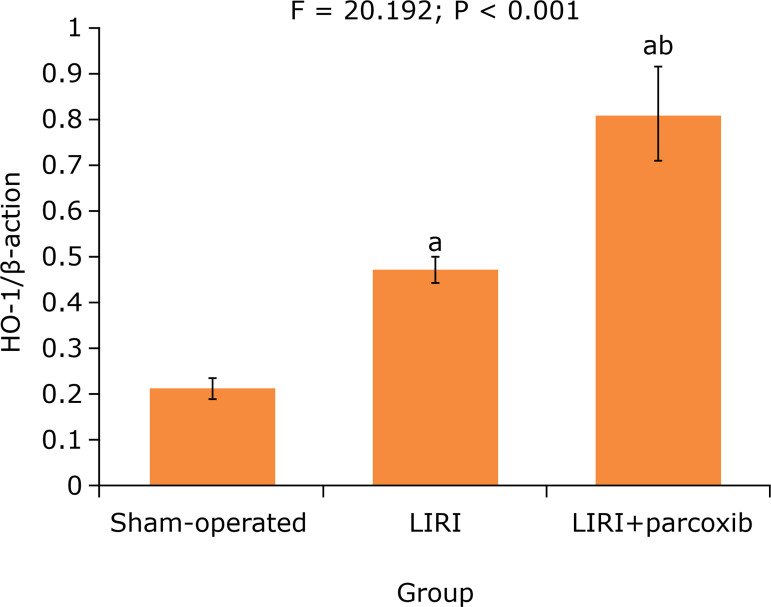
Comparison of lung tissue HO-1 protein expression level in three groups (n = 10).

^a^P < 0.05 *vs*. sham-operated group; ^b^P < 0.05 *vs*. LIRI group; LIRI: lung ischemia-reperfusion injury; HO-1: heme oxygenase-1.

After 120 min of reperfusion, the western blotting assay showed that the lung tissue HO-1/ß-actin ratio in sham-operated, LIRI and LIRI+parecoxib groups was 3.82±0.48, 5.79±0.92 and 4.55±0.63, respectively. The HO-1/ß-actin ratio in LIRI and LIRI+parecoxib groups was higher than that in sham-operated group (P < 0.05), and that in LIRI+parecoxib group was higher than that in LIRI group (P < 0.05) (Fig. 2).

## Discussion

After LIRI, the alveolar capillary barrier is destroyed, the permeability of lung tissue is increased, and the exudation of plasma components was increased. It causes the pulmonary interstitial edema and bleeding, leading to the ventilation dysfunction. The lung wet to dry mass ratio can indirectly reflect the degree of pulmonary interstitial edema. OI and PA-aO_2_ can be used as indicators to judge whether the lung ventilation function is normal. It is found that parecoxib can attenuate the hepatic, myocardial and cerebral ischemia-reperfusion injury^6^,^11^,^12^.

In the present study, the protective effect of parecoxib against LIRI in rats was investigated. Results showed that, after 120 min of reperfusion, compared with sham-operated group, in LIRI and LIRI+parecoxib groups the OI was significantly decreased, the PA-aO_2_ was significantly increased, and the lung wet to dry mass ratio was significantly increased. This suggests that there are pulmonary interstitial edema and lung ventilation function abnormality in LIRI rats. Compared with LIRI group, above indexes in LIRI+parecoxib group were significantly improved. It indicates that the parecoxib pretreatment can reduce the pulmonary interstitial edema and enhance the lung ventilation function in LIRI rats.

The production of free radicals is an important factor of ischemia-reperfusion injury^13^. MDA is a degradation product of unsaturated fatty acids in lipid peroxidation by oxygen free radicals. It can be used as an important marker to judge the production of oxygen free radicals and tissue damage^14^. SOD is the main oxygen free radical scavenger, and its activity reflects the antioxidant capacity of the body^15^. The activated neutrophils are an important source of oxygen free radicals, and the MPO activity is the decisive factor in neutrophil oxidation^16^.

It is found that parecoxib can resist the oxidative stress in body^17^. Results of the present study showed that, compared with sham-operated group, in LIRI and LIRI+parecoxib groups the lung tissue MDA content was significantly increased, and the lung tissue SOD and MPO activities were significantly decreased. Compared with LIRI group, in LIRI+parecoxib group the lung tissue MDA content was significantly decreased, and the lung tissue SOD and MPO activities were significantly increased. This suggests that the oxidative stress is involved in the LIRI in rats, and the parecoxib pretreatment can reduce the oxidative stress, thus alleviating the LIRI.

Excessive inflammatory response plays an important role in the pathological process of LIRI. Many cells participate in the process of LIRI, leading to inflammatory cell infiltration and release of a large number of cytokines such as TNF-a, IL-1ß and others^1^. TNF-a is a cytokine with a wide range of biological functions. IL-1ß can bind to the TNF-a receptor, and induce its transcription^18^,^19^. The over-expression of TNF-a and IL-1ß can promote the neutrophil aggregation and chemotaxis, and increase the vascular permeability. This results in the pulmonary interstitial edema, which affects the gas exchange and aggravates the lung injury^20^.

Previous studies^21^,^22^ have shown that parecoxib have the anti-inflammatory effect. In this study, the lung tissue TNF-a and IL-1ß levels in LIRI and LIRI+parecoxib groups were significantly higher than those in sham-operated groups. Compared with LIRI group, each index in LIRI+parecoxib group was significantly decreased. This indicates that the alleviation of LIRI by parecoxib pretreatment may be related to its inhibition of inflammatory response in lung tissue.

HO-1 is a rate-limiting enzyme of heme metabolism in mammalian cells and can catalyze the degradation of free heme to iron, carbon monoxide and biliverdin and reduce the production of oxygen free radicals by degrading free heme in damaged cells^23^. In addition, carbon monoxide, another metabolite of heme, has the anti-inflammatory, anti-proliferative and apoptotic effects^24^. It has been found that the increased expression of HO-1 can protect lung tissue from ischemia-reperfusion injury^25^. In the present study, after reperfusion, the lung tissue HO-1 protein expression level in LIRI and LIRI+parecoxib groups was higher than that in sham-operated group, and that in LIRI+parecoxib group was higher than that in LIRI group. This suggests that the parecoxib pretreatment can up-regulate the HO-1 expression in lung tissue, thus alleviating the LIRI in rats.

## Conclusion

The parecoxib pretreatment can reduce the pulmonary interstitial edema and enhance the lung ventilation function in LIRI rats. The mechanism may be related to its reducing oxidative stress, inhibiting inflammatory response and up-regulating HO-1 expression in lung tissue.

This study still has some limitations. Firstly, only one dose of parecoxib was investigated, and the dose-effect relationship is relatively simple. Secondly, this study has not investigated other action mechanism of parecoxib. In next studies, the dose-effect relationship of parecoxib and the other action mechanisms of parecoxib on LIRI should be further explored.
